# Clinical Features and Characteristics of Hand, Foot, and Mouth Disease Caused by Recent Coxsackievirus A6: Five Cases in Japan from 2019 to 2022

**DOI:** 10.3390/idr16040044

**Published:** 2024-06-29

**Authors:** Kyohei Naomiya, Takashi Ito, Ayumi Saito, Tsukasa Igarashi, Tetsuo Nakayama, Kazuhiko Katayama, Kenji Ishikura

**Affiliations:** 1 Department of Pediatrics, Kitasato University School of Medicine, 1-15-1 Kitasato, Minami-ku, Sagamihara City 252-0329, Japan; 2 Ōmura Satoshi Memorial Institute, Kitasato University, 5-9-1 Shirokane, Minato-ku, Tokyo 108-8641, Japan

**Keywords:** enterovirus, coxsackievirus A6, hand, foot, and mouth disease, herpes zoster virus, herpes simplex virus, reverse transcription–polymerase chain reaction (RT-PCR)

## Abstract

Hand, foot, and mouth disease (HFMD) is a common infectious disease caused by enteroviruses. Coxsackievirus A6 (CV-A6)-associated HFMD has recently emerged as a predominant disease worldwide. Here, we describe five HFMD cases caused by CV-A6 in Japan from 2019 to 2022. All clinical courses were not severe and were self-limited, and the skin exanthema with vesicles differed from that in classical HFMD. Phylogenetic analysis showed that the major epidemic strain cluster of CV-A6 was formed independently in 2011, and our latest CV-A6 strains in Japan were detected within this cluster. The five cases described in this report indicate the recent shift in the predominant and continuous disease manifestation of CV-A6-associated HFMD.

## 1. Introduction

Hand, foot, and mouth disease (HFMD) is a common infectious disease caused by enteroviruses [1] and characterized by vesicular lesions in the oral cavity and on the hands and feet, accompanied by fever [2]. To date, more than 20 types of enteroviruses causing HFMD have been identified, and the major serotypes are enterovirus A71 (EV-A71) and coxsackievirus A16 (CV-A16) of enterovirus species A [3]. Atypical CV-A6-associated HFMD cases were first reported in 2008 [4] and have subsequently been reported with increasing frequency worldwide [5,6,7]. CV-A6 is a single-stranded RNA virus belonging to the family Picornaviridae and the genus enterovirus [5]. CV-A6 has recently emerged as the predominant circulating enterovirus worldwide [6,7], including in Japan [8]. Compared with classical HFMD caused by EV-A71 and CV-A16, CV-A6-associated HFMD is characterized by extensive exanthema with vesicles all over the body [1].

Here, we describe the clinical features and viral characteristics of five HFMD cases caused by CV-A6 in Japan from 2019 to 2022. We also conduct a phylogenetic analysis using the complete length of the nucleotide sequence of the open reading frame and discuss the changes in pathogenicity.

## 2. Materials and Methods

### 2.1. Study Participants

In this retrospective study of case series, skin vesicular, pharyngeal swab, and stool samples were collected from inpatients with fever or skin rash symptoms at Kitasato University Hospital and Sagamihara Kyodo Hospital between August 2019 and December 2022. During this period, 37 HFMD cases were positive for enterovirus in the first real-time polymerase chain reaction (PCR) assay, and 11 cases (29.7%) were infected with CV-A6. We clinically selected five characteristic cases for this study.

### 2.2. Viral Detection and Sequence

Total RNA was extracted using an RNeasy Plus Mini Kit (Qiagen, Gaithersburg, MD, USA). First, real-time reverse transcription PCR (RT-PCR), which targeted the conserved regions of the 5′ noncoding region, was performed as previously reported [9]. Next, consensus-degenerate hybrid oligonucleotide primer (CODEHOP)-RT-PCR was performed to determine the partial sequence of the capsid protein viral protein 1 (VP1) coding region [10]. Direct sequence determination led to subsequent identification as CV-A6, using the National Institute for Public Health and the Environment (RIVM) systems [11]. cDNA was generated using a PrimeScript IV First-Strand Synthesis system (TaKaRa Bio, Shiga, Japan) to determine the full-length sequences, and negative-sense gene-specific primers were generated based on the conserved regions. PCR primers were designed to amplify the complete viral genome in multiple small overlapping fragments. The sequences of the genome end were determined by the rapid amplification of cDNA ends. The leader sequence was determined by directly A-tailing the viral RNA using poly A polymerase (New England Biolabs, Ipswich, MA, USA) followed by first-strand cDNA synthesis. The trailer sequence was determined by synthesizing first-strand cDNA, which was A-tailed using terminal transferase (New England Biolabs, Ipswich, MA, USA) followed by PCR. Full-length sequences were registered in the DNA Data Bank of Japan. Phylogenetic analysis was conducted using the Bayesian Markov Chain Monte Carlo method (Bayesian MCMC), and time-scaled phylogeny was determined using the BEAST program package v2.6.7. To estimate branch ages in molecular phylogenetic analysis, we analyzed 32 previously reported CV-A6 sequences with documented isolation years from the database, in addition to our five clinical cases. Total RNA extraction, PCR analysis, and sequencing of the VP1 coding region were performed at Kitasato University pediatrics laboratory (clinical lab.), and full-length sequencing and phylogenetic analysis were conducted at Kitasato University, Ōmura Satoshi Memorial Institute (viral basic lab.).

### 2.3. Ethics Statement

This study complied with the Declaration of Helsinki and was approved by the ethics review board of Kitasato University Hospital (No. B21-246). Informed consent was obtained from the parents of each patient (cases 1–4) or the patient (case 5) included in this study.

## 3. Results

### 3.1. Cases

**Figure 1:**
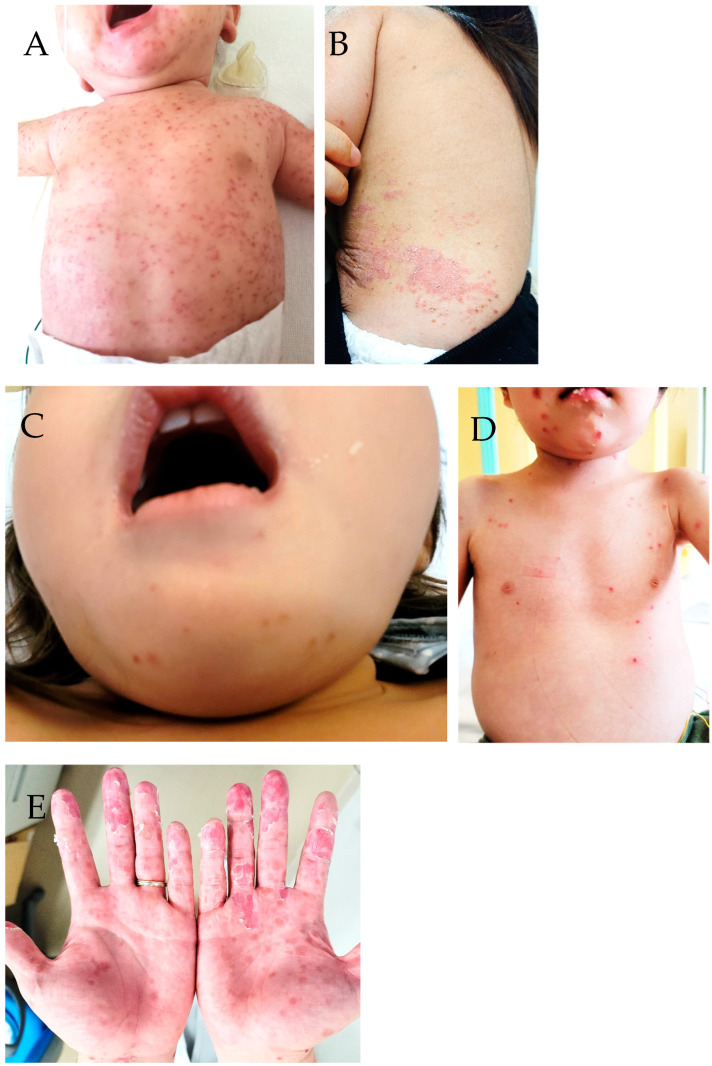
(**A**). Rash covering the entire face, body trunk, and upper extremities of the 2-month-old boy in case 1. (**B**). Rash on the lower back of the healthy 1-year-old girl in case 2; some of them were fused and crusted. (**C**). Rash around the mouth of the 2-year-old girl in case 3. (**D**). Rash on the face and body trunk of the 3-year-old boy in case 4. (**E**). Rash on the hands of the 34-year-old man in case 5; parts of the epidermis of the fingertips were exfoliated.

- Case 1: A 2-month-old boy was hospitalized due to pyrexia and systemic exanthema in August 2019. On admission, his body temperature was 38.5 °C and he had a slight reddish rash with vesicles on his entire face, body trunk, back, and upper and lower extremities, excluding his palms and soles (Figure 1A). Clinical specimens of pharyngeal swabs and vesicular fluid were positive for CV-A6 (accession number: LC715423 from skin sample). Three days after admission, his fever subsided, and he was discharged on day seven of hospitalization. The systemic exanthema disappeared gradually and completely disappeared one month after discharge.
- Case 2: A 1-year-old girl, generally healthy, was presented to the hospital with a belt-like confluent rash around her abdomen and thighs in December 2021 (Figure 1B). After 2 days, the rash spread over the entire body. During consultation, her general condition was stable, with a temperature of 36.7 °C. Blood tests revealed no abnormalities, whereas RT-PCR of the skin vesicular fluid revealed a positive result for CV-A6 (accession number: LC715248). The rash and vesicles disappeared after 13 days.
- Case 3: A 2-year-old girl was hospitalized due to febrile convulsions in September 2022. Her body temperature was 38.5 °C, and she developed reddish papules with a few vesicles on her upper and lower extremities and around the mouth (Figure 1C). No abnormal findings were detected in blood tests, spinal fluid tests, or head computed tomography scans. CV-A6 was detected in skin and pharyngeal swab specimens (accession number: LC789937 from skin sample). After 2 days of hospitalization, her temperature decreased to 36.1 °C, and no further convulsions occurred. She was discharged on day 5, and the rash disappeared after 10 days. No symptoms of encephalitis or encephalopathy were observed, and the diagnosis of this case was simple febrile convulsion.
- Case 4: A 3-year-old boy who was followed up for autoimmune lymphoproliferative disease (with genetic mutations) and was on immunosuppressive drugs and steroids developed a fever up to 38.5 °C in September 2022. The next day, small papules appeared on the bilateral sides of his forearms and buttocks, and the rash intensified. On examination, there were several blisters in the oral cavity, numerous erythematous vesicular papules on the scalp, face, anterior chest, extremities, and buttocks, and some blisters (Figure 1D). He was hospitalized with suspected chickenpox. Two days after admission, numerous vesicles and blisters distributed throughout the body. Pharyngeal and skin swabs were positive for CV-A6 (accession number: LC789938 from skin sample), negative for varicella zoster virus (VZV) in the RT-PCR assay, and serum IgM-negative for VZV. After 5 days, he was discharged because the fever had reduced, and the rash had receded. The rash disappeared entirely after 1 month.
- Case 5: A 34-year-old man had a fever of 39 °C with edema and erythema of the palms and small papules on the lower legs (calves) in August 2022. His fever resolved after 2 days, but the other symptoms intensified. Palmar redness and partial desquamation were detected after 5 days (Figure 1E). The palm skin and stool samples were positive for CV-A6 (accession number: LC789939 from stool sample) in the RT-PCR assay. Papules on the lower legs gradually improved and disappeared after 10 days, palm erythema disappeared after 14 days, and desquamation disappeared after 1 month.

### 3.2. Phylogenetic Analysis

**Figure 2:**
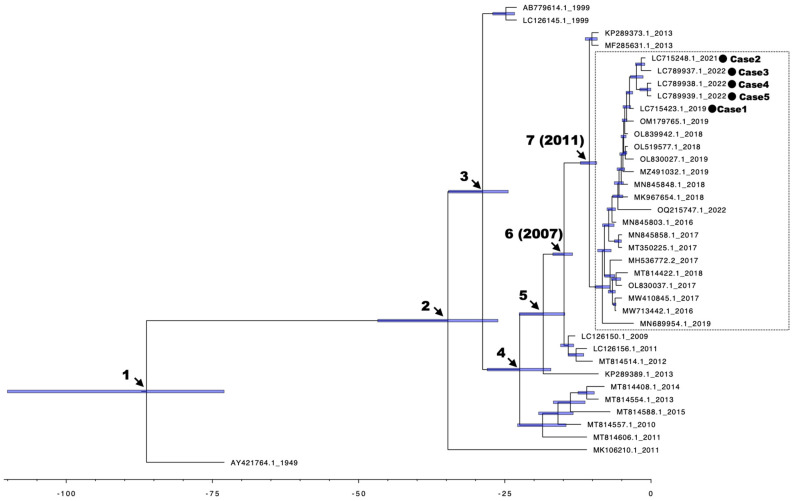
The phylogenetic tree of complete ORF constructed using the Bayesian Markov Chain Monte Carlo method. The phylogenetic tree was based on the whole nucleotide sequence of ORF (6606 nt corresponding to ICTV standard CV-A6: AY421764). We analyzed 37 strains of CV-A6. Each node represents mean root height. The scale bar indicates the years before 2022 as negative numbers, considering 2022 as the reference point (zero). The time unit is in years. The purple thick horizontal bars represent the 95% highest Posterior Density interval for the estimated years. The strain names are expressed with accession numbers and the year of sample collection. The numbers indicate branching events. Dashed squares represent clusters of CV-A6 sampled after 2016.

We conducted a Bayesian MCMC analysis using 37 CV-A6 strains, which revealed five branching events from the ancestral strain AY421764 (Figure 2). In the sixth branching event in 2007, a cluster containing strains that underwent a pathogenic shift from herpangina to HFMD emerged. Subsequently, in the seventh branching event in 2011, a major epidemic strain cluster was formed, which has been detected in patients with HFMD worldwide since 2016. Our five cases were positioned within this cluster.

## 4. Discussion

Here, we describe five HFMD cases caused by CV-A6. As reported previously [12], it is important to clinically distinguish CV-A6-associated HFMD from other viral infections, such as Kaposi’s varicelliform eruption, varicella, herpes zoster, adenovirus infections, and others. In fact, cases 1 and 4 were suspected to have varicella and/or severe herpes simplex infection, and case 2 was thought to be herpes zoster and/or heat rash. Case 3 presented with febrile convulsion. Although neurological complications, including viral meningitis and encephalitis, of CV-A6-associated HFMD are uncommon compared with classical HFMD [13], a previous report suggested severe neuroinfectious diseases such as encephalomyelitis [14]. Case 5 was an adult case. HFMD is commonly observed in children aged < 5 years; however, clusters of adult cases have also been reported in CV-A6-associated HFMD [15]. Similar to this case, CV-A6-associated HFMD is characterized by palmoplantar desquamation after 1–3 weeks [16].

In Japan, atypical HFMD cases caused by CV-A6 have been reported every other year from 2011 to 2019, and most HFMD cases were mild [17]. Although the clinical characteristics of the five HFMD cases in this study were not novel and were similar to those of previous reports, we demonstrated by phylogenetic analysis that the major epidemic strain cluster of CV-A6 formed independently in 2011, and our latest CV-A6 strains in Japan were detected within this cluster. These data indicate the genetic evolutionary pathway of CV-A6 consistent with clinical HFMD symptoms at least after 2011 in Japan. The five cases described in this report indicate the recent shift in the predominant and continuous disease manifestation of CV-A6-associated HFMD.

## 5. Conclusions

We documented five CV-A6-associated HFMD cases. All clinical courses were not severe and were self-limiting, and the skin rash and vesicles differed from typical HFMD manifestations. The shift in the predominant disease manifestation of CV-A6 from herpangina and typical HFMD to the atypical HFMD described in this report suggests a changing pattern in CV-A6 pathogenesis.

## Data Availability

The anonymized data underlying the results presented in this manuscript may be made available to researchers upon submission of a reasonable request to the corresponding author. The decision to disclose the data will be made by the corresponding author.
